# Using CRISPR/Cas9 to identify genes required for mechanosensory neuron development and function

**DOI:** 10.1242/bio.060002

**Published:** 2023-09-05

**Authors:** Christopher J. Johnson, Akhil Kulkarni, William J. Buxton, Tsz Y. Hui, Anusha Kayastha, Alwin A. Khoja, Joviane Leandre, Vanshika V. Mehta, Logan Ostrowski, Erica G. Pareizs, Rebecca L. Scotto, Vanesa Vargas, Raveena M. Vellingiri, Giulia Verzino, Rhea Vohra, Saurabh C. Wakade, Veronica M. Winkeljohn, Victoria M. Winkeljohn, Travis M. Rotterman, Alberto Stolfi

**Affiliations:** School of Biological Sciences, Georgia Institute of Technology, 30332 Atlanta, GO, USA

**Keywords:** Ascidian, CRISPR/Cas9, Ciona, Education, Marine larvae, Tunicate

## Abstract

Tunicates are marine, non-vertebrate chordates that comprise the sister group to the vertebrates. Most tunicates have a biphasic lifecycle that alternates between a swimming larva and a sessile adult. Recent advances have shed light on the neural basis for the tunicate larva's ability to sense a proper substrate for settlement and initiate metamorphosis. Work in the highly tractable laboratory model tunicate *Ciona robusta* suggests that sensory neurons embedded in the anterior papillae transduce mechanosensory stimuli to trigger larval tail retraction and initiate the process of metamorphosis. Here, we take advantage of the low-cost and simplicity of *Ciona* by using tissue-specific CRISPR/Cas9-mediated mutagenesis to screen for genes potentially involved in mechanosensation and metamorphosis, in the context of an undergraduate ‘capstone’ research course. This small screen revealed at least one gene, *Vamp1/2/3*, which appears crucial for the ability of the papillae to trigger metamorphosis. We also provide step-by-step protocols and tutorials associated with this course, in the hope that it might be replicated in similar CRISPR-based laboratory courses wherever *Ciona* are available.

## Teaser

**Summary:** Using CRISPR/Cas9 to study neuronal function and development in a classroom context.

## INTRODUCTION

Solitary tunicates (*Ciona spp.*) have emerged as highly tractable model organisms for developmental, cell, and molecular biology (Cota, 2018; Lemaire, 2011). Tissue-specific CRISPR/Cas9-mediated mutagenesis has been adapted to *Ciona robusta* and is now routinely employed to test the functions of genes in *Ciona* embryos and larvae (Gandhi et al., 2018; Sasakura and Horie, 2023). The low-cost and ease of CRISPR/Cas9 in *Ciona* makes these animals ideal organisms for laboratory courses in higher education. Hands-on experience in CRISPR/Cas9 might prepare students for a world in which CRISPR/Cas9-based technologies become more prevalent (Thurtle-Schmidt and Lo, 2018).

**Fig. 1.**
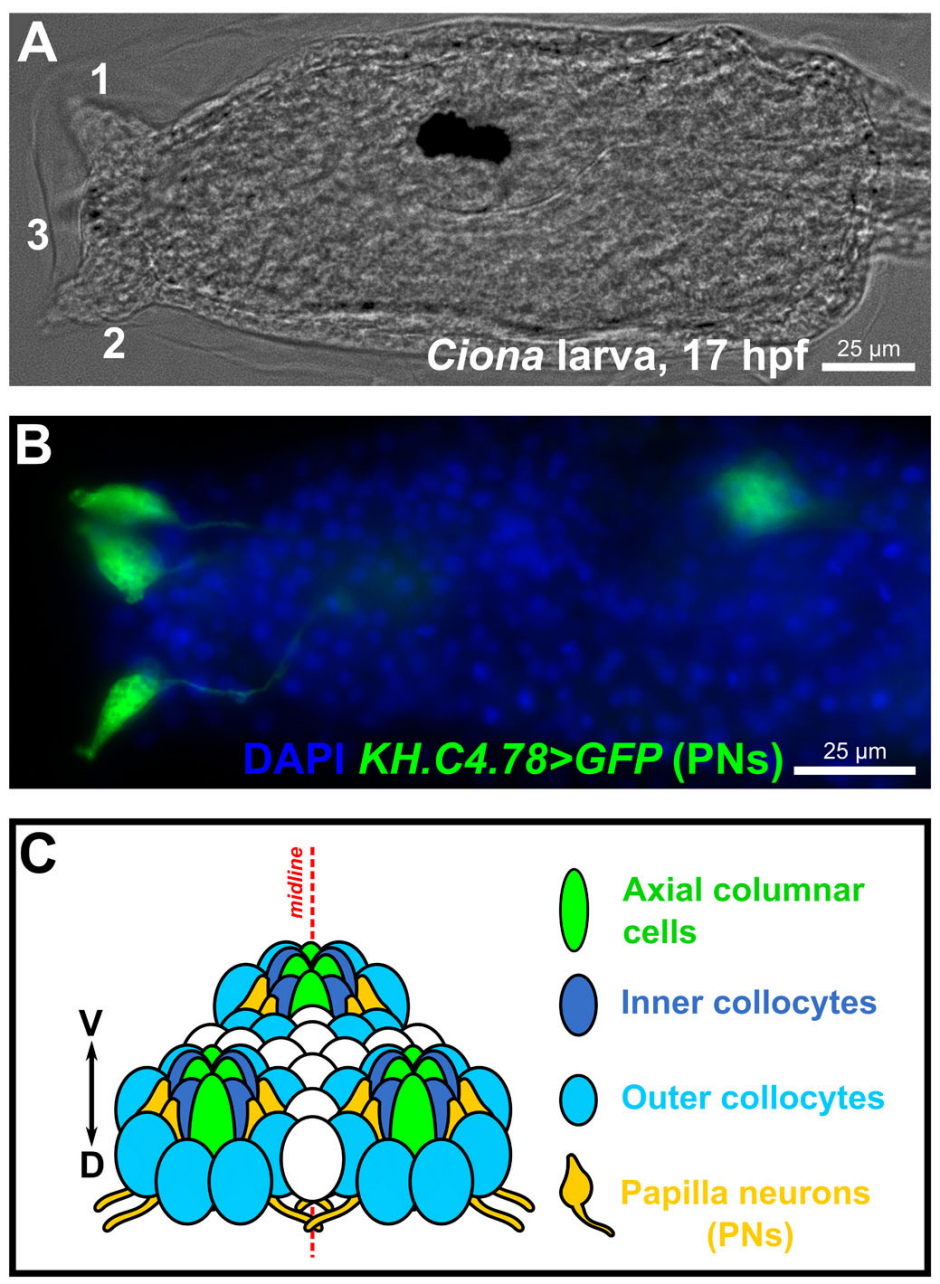
**The sensory/adhesive papillae of the *Ciona* larva.** (A) Brightfield image of a *Ciona robusta* (*intestinalis* type “A”) larva at 17 hpf raised at 20°C, showing the three protruding papillae of the head (numbered 1-3). Papilla number 3, the medial/ventral papilla, is out of focus. (B) Image of electroporated *Ciona* larva at 17 hpf/20°C, papilla neurons (PNs) labeled by the reporter plasmid *KH.C4.78>Unc-76::GFP* (green, from Johnson et al., 2023 preprint). Nuclei counterstained by DAPI (blue). (C) Summary diagram of the arrangement and cell type diversity of the papillae (from Johnson et al., 2023 preprint).

Here, we used *Ciona robusta* in the context of an undergraduate ‘capstone’ research course on the use of CRISPR/Cas9 in neurobiology, taught at the Georgia Institute of Technology. In this course, students selected four target genes from a list of genes putatively expressed in the mechanosensory neurons of the anterior papillae of the *Ciona* larvae. The papillae are a group of three small clusters of cells organized in a triangle at the anterior end of the larval head (Fig. 1). Basic characterization of the cell types contained in these papillae suggest multiple adhesive, contractile, and sensory functions supporting the attachment of the larvae to the substrate and triggering the onset of metamorphosis (Nakayama-Ishimura et al., 2009; Zeng et al., 2019a,b). Recently, mechanical stimulus of the papillae was shown to be sufficient for triggering tail retraction, the first stage of metamorphosis (Wakai et al., 2021). This ability was shown to depend on PKD2-expressing papilla neurons specified by the transcription factor Pou4 (Sakamoto et al., 2022).

With this in mind, students in the course hypothesized that one or more genes expressed in the papillae neurons might be required for tail retraction and metamorphosis. Students designed and validated single-chain guide RNAs (sgRNAs) targeting four selected genes: *Tyrosine hydroxylase (TH)*, *Vamp1/2/3*, *Neuronal calcium sensor 1 (NCS1)*, and *NARS1*. Of these, *Vamp1/2/3* was the only gene that, when knocked out, resulted in a metamorphosis defect. However, *NARS1* knockout in the developing central nervous system resulted in major morphological defects, indicating that our validated sgRNAs might still be instrumental in revealing the roles of these genes in other contexts. Here we describe our findings, in addition to providing detailed sequence information and protocols. We hope that this study will help other instructors who wish to implement a similar lab course based on CRISPR and/or *Ciona*, or researchers who wish to knock out these same *Ciona* genes out in other cell types.

## RESULTS

### Selecting genes and designing sgRNAs

Genes to be targeted by CRISPR/Cas9 were chosen based on student preference, from a list of transcripts enriched in a cell cluster potentially representing the papilla mechanosensory neurons, identified from whole-larva singe-cell RNA sequencing data. Briefly, published data (Cao et al., 2019) were reanalyzed (Johnson et al., 2023 preprint) and papilla neuron identity was tentatively confirmed by enrichment with *Thymosin beta-related (KH.C2.140)*, *Celf3/4/5 (KH.C6.128)*, *Foxg (KH.C8.774)*, *Synaptotagmin (KH.C2.101)*, *Pou4 (KH.C2.42)*, *Pkd2 (KH.C9.319)*, and *TGFB (KH.C3.724)*, based on previous reports (Horie et al., 2018; Katsuyama et al., 2002; Razy-Krajka et al., 2014; Sakamoto et al., 2022; Sharma et al., 2019; Zeng et al., 2019b) (Table S1, additional files found at https://osf.io/sc7pr/). To be clear, these are distinct from what we previously called ‘palp neurons’ (Sharma et al., 2019), which were later identified conclusively as a non-neuronal cell type, the Axial Columnar Cells of the papillae (Johnson et al., 2020; Zeng et al., 2019b). The genes and sgRNAs selected for this study are detailed below.

### Tyrosine hydroxylase (KH.C2.252)

**Fig. 2.**
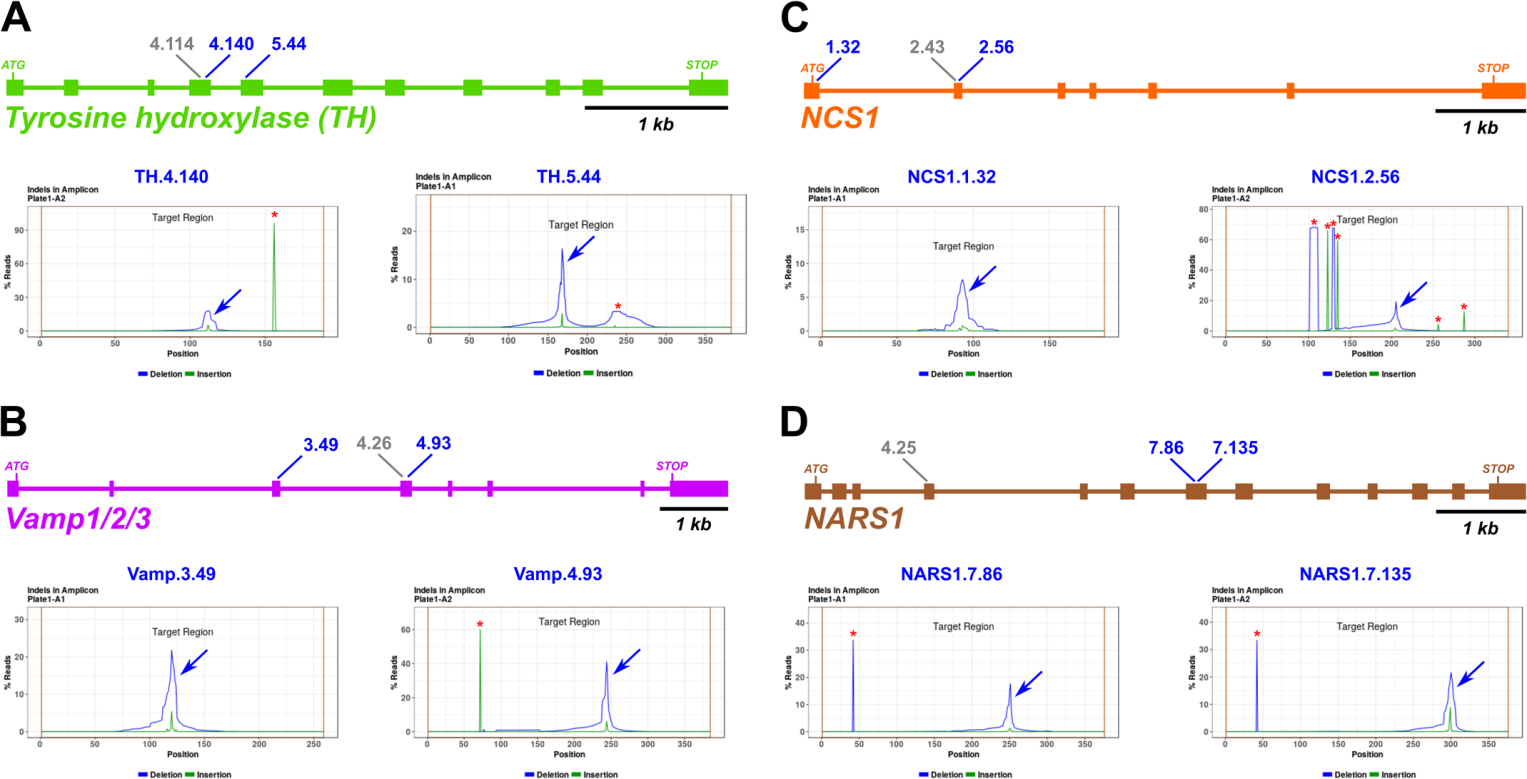
**Design and validation of sgRNAs for CRISPR/Cas9-mediated mutagenesis.** (A-D) Diagrams of selected candidate gene loci and indel analysis plot for each selected sgRNA, based on next-generation sequencing of amplicons. Blue arrows indicate CRISPR/Cas9-induced indel peak, red asterisks indicate naturally-occurring indels. Blue sgRNA identifiers indicate top sgRNAs selected for phenotypic assay. Grey identifiers indicate sgRNA designed, tested, but not selected for further use.

The gene selected by the first group of students was *Tyrosine hydroxylase* (*TH;* KyotoHoya gene model ID: *KH.C2.252*)*,* encoding the *C. robusta* ortholog of the rate-limiting enzyme of dopamine biosynthesis (Moret et al., 2005). Previously, *TH* was reported to be a marker of putative dopamine-releasing coronet cells of the ventral larval brain vesicle (Moret et al., 2005; Razy-Krajka et al., 2012; Takamura et al., 2010). Dopamine immunoreactivity was also observed in the papilla region of another species, *Phallusia mammillata* (Zega et al., 2005). Pharmacological treatments suggested roles for dopamine in neuromodulation of tail muscle contractions in *Ciona* (Razy-Krajka et al., 2012), and suppression of metamorphosis in *P. mammillata* (Zega et al., 2005). More recently, a ‘beat-and-glide’ behavior of *Ciona* larvae was found to be affected by pharmacological inhibition of dopamine and other monoamine neurotransmitters (Athira et al., 2022). Three sgRNAs were selected from those predicted by the web-based CRISPOR prediction tool (crispor.tefor.net) (Haeussler et al., 2016), as described in detail in the methods section and online protocols. Two were predicted to cut in exon 4 (named “TH.4.114” and “TH.4.140”) and one in exon 5 (“TH.5.44”) (Fig. 2A). Because exon 5 encodes the beginning of the major catalytic domain of TH, these sgRNAs were predicted to generate frameshift mutations resulting in truncated proteins lacking the catalytic domain.

### Vamp1/2/3 (KH.C1.165)

The second student group picked *Vamp1/2/3* (*KH.C1.165*), which encodes a member of the synaptobrevin family of SNARE complex proteins that carry out neurotransmitter vesicle release (Rizo, 2022). Based on phylogenetic analysis in MAFFT (see Materials and Methods), *Vamp1/2/3* (*KH.C1.165*) appears to be orthologous to *VAMP1*, *VAMP2*, and *VAMP3* in humans (Fig. S1). Its potentially evolutionarily conserved function and broad expression in the *Ciona* larval nervous system suggested an important role for *Vamp1/2/3* in neurotransmitter release in *Ciona,* including in the papilla neurons during settlement. The *Vamp1/2/3* gene in *Ciona* appears to give rise to a few different alternatively spliced isoforms. The sgRNAs selected from CRISPOR included one sgRNA targeting exon 3 (“Vamp.3.49”) and two sgRNAs targeting exon 4 (“Vamp.4.26” and “Vamp.4.93”) in the “v3” and “v4” transcript variants (Fig. 2B). These exons become exons 2 and 3, respectively, in all other transcript variants.

### Neuronal calcium sensor 1 (KH.C1.1067)

Group number 3 selected the gene *Neuronal calcium sensor 1* (*NCS1,* gene model *KH.C1.1067*)*.* According to our phylogenetic analysis, KH.C1.1067 appeared to be most similar to human NCS1 and its *Drosophila melanogaster* orthologs, Frequenin1 and Frequenin2 within the NCS family of proteins (Fig. S2A). NCS1/Frq proteins regulate neurotransmission through both pre- and post-synaptic mechanisms (Dason et al., 2012), likely on account of their ability to bind Ca2+ ions through their multiple EF hand domains. In *Ciona*, *NCS1* had been previously identified as a transcriptional target of Neurogenin in the Bipolar Tail Neurons of the larva, suggesting a broader role in neuronal function (Kim et al., 2020). However, no function has yet been shown for this gene in *Ciona*. Three sgRNAs targeting *NCS1* were selected for testing: one sgRNA targeting exon 1 (“NCS1.1.32”) and two sgRNAs targeting exon 2 (“NCS1.2.43” and “NCS1.2.56”) (Fig. 2C). As these sgRNAs are predicted to cut 5′ to the exons encoding the EF hand domains (exons 3-7, Fig. S2B), the resulting frameshift mutations are predicted to result in a truncated, non-functional polypeptide.

### NARS1 (KH.C12.45)

The fourth student group picked *NARS1* (*KH.C12.45*), which encodes the *C. robusta* ortholog of Aparaginyl tRNA synthetase 1 (cytoplasmic), which catalyzes the attachment of asparagine (Asn/N) to its cognate tRNAs (Shiba et al., 1998). In a neurodevelopmental context, it has been shown that loss of *NARS1* in human brain organoids impairs neural progenitor proliferation (Wang et al., 2020). Mutations in *NARS1* is associated with various neurodevelopmental syndromes such as microcephaly and cognitive delays (Wang et al., 2020), suggesting that regulation of protein synthesis rates is indispensable for development of the nervous system. Phylogenetic analysis shows that these aminoacyl-tRNA synthetases are highly conserved in their specificity, with simple 1-to-1 orthology between *Ciona* and human genes of various types and classes within this gene family (Fig. S3A). For this gene, one sgRNA targeting exon 4 (“NARS1.4.25”) and two sgRNAs targeting exon 7 (“NARS1.7.86” and “NARS1.7.135”) were designed (Fig. 2D). While the tRNA anti-codon domain is encoded by exons 5-7, and the tRNA synthetase domain is encoded by exons 7-13, these sgRNAs are predicted to result in truncated NARS1 polypeptides lacking both major functional domains (Fig. S3B).

### Validation of sgRNA efficacy by Illumina amplicon sequencing

**Fig. 3.**
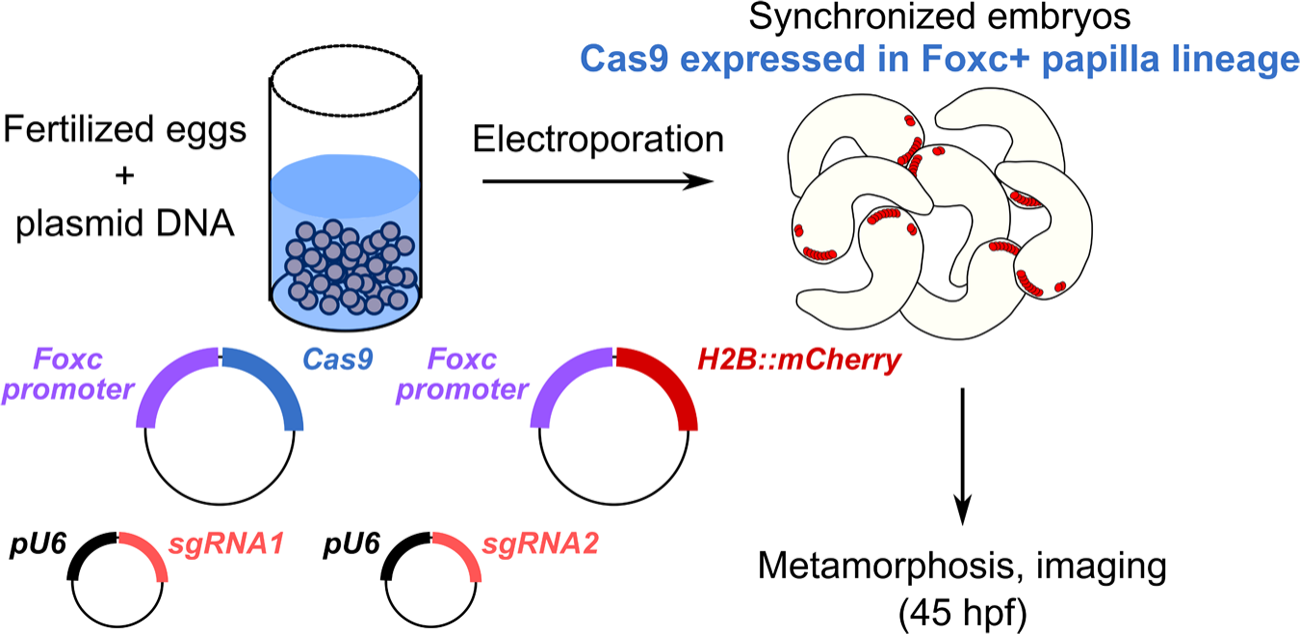
**Tissue-specific CRISPR/Cas9-mediated mutagenesis for tail retraction assay.** Briefly, synchronized zygotes are transfected with plasmids encoding Cas9, sgRNAs, and fluorescent reporters, and resulting embryos are fixed for imaging at 45 hpf, during metamorphosis.

Validation of sgRNA efficacies was performed by sequencing amplicons surrounding each target site, from larvae electroporated with a given sgRNA vector together with the ubiquitously expressed *Eef1a>Cas9* (Stolfi et al., 2014)(Fig. 3). Although we had previously reported a Sanger sequencing-based method for estimating mutagenesis efficacy (Gandhi et al., 2018), that strategy is frequently hampered by naturally occurring indels and poor sequencing quality. We decided instead to quantify mutagenesis by sequencing amplicons using a commercially available Illumina-sequencing based service, as recently described (Johnson et al., 2023 preprint).

Briefly, 75 µg/700 µl total electroporation volume of each sgRNA plasmid was co-electroporated with 25 µg/700 µl of *Eef1a>Cas9* into zygotes, which were collected at larval stage (∼17 h post-fertilization, reared at 20°C). Larvae electroporated with the same sgRNA vector were pooled, and genomic DNA extracted from them. DNA fragments spanning each target site, ranging from 150-450 bp as required by the sequencing service, were amplified by PCR from each genomic DNA pool. Negative controls for each amplicon were derived by repeating the PCR on samples from larvae electroporated with sgRNA vector targeting a different sequence (e.g. targeting exon 2 instead of exon 3). Amplicons were then submitted for library preparation, sequencing, and analysis by Genewiz/Azenta. The sgRNAs chosen for further experiments were those that resulted in a larger portion of on-target indels based on visual examination of the indel plot automatically generated by the amplicon sequencing service. This was better than relying on raw mutagenesis rates provided by the service, as the plots revealed a high frequency of naturally occurring indels that prevented the automatic quantification of the true efficacies of some sgRNAs. This new approach is described in greater detail in the methods and online protocols.

Amplicon sequencing revealed the indels induced by all sgRNAs except for NARS1.4.25, which was not evaluated due to failure to amplify its target site by PCR (Fig. 2, Fig. S4). For *TH*, we found that all three sgRNAs were effective at generating indels at the target sequences, though TH.4.140 and TH.5.44 were selected, as having barely edged out TH.4.114 (Fig. 2A, Fig. S4A). For *Vamp1/2/3*, the most efficient sgRNAs was Vamp.4.93 (>40% efficacy, Fig. 2B), while Vamp.3.49 and Vamp.4.26 were less efficacious at ∼20-30% indels (Fig. S4B). Because we wished to use a pair of sgRNAs targeting different exons, we selected Vamp.4.93 and Vamp.3.49 for further use. All three sgRNAs targeting *NCS1* generated indels, though NCS1.1.32 efficacy was only 12% indels (Fig. 2C, Fig. S4C). Because NCS1.2.43 and NCS1.2.56 targets overlapped, we paired the most efficacious sgRNA (NCS1.2.56) with NCS1.1.32. Finally, NARS1.7.86 and NARS1.7.135 resulted in mutagenesis efficacy rates >15% (Fig. 2D). As these were the only two *NARS1-*targeting sgRNAs for which amplicons were successfully amplified by PCR (Fig. S4D), we proceeded with both and did not further use the untested sgRNA NARS1.4.25.

### Papilla lineage-specific knockout of target genes by CRISPR/Cas9

**Fig. 4.**
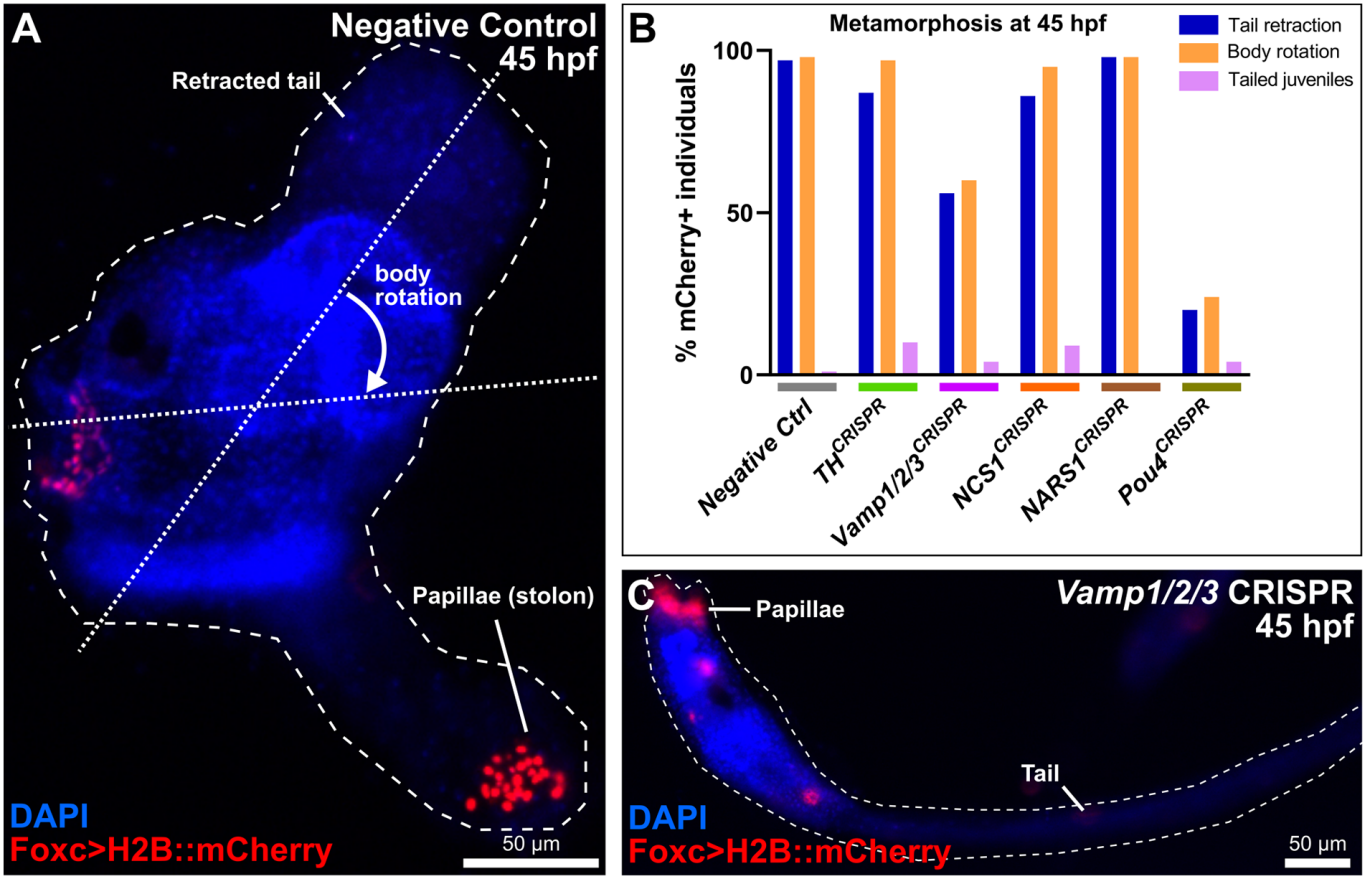
**Scoring metamorphosis defects in CRISPR larvae.** (A) Example of a juvenile at 45 hpf in the “negative control” population, showing the retracted tail and body rotation that occurs during metamorphosis. *Foxc>H2B::mCherry* (red) labels the cells of the oral siphon and the papillae, the latter of which are transformed into the stolon of the juvenile. Nuclei counterstained by DAPI (blue). (B) Scoring of *Foxc>H2B::mCherry+* individuals upon papilla-specific CRISPR/Cas9-mediated mutagenesis of the selected candidate genes. “Tailed juveniles” are individuals that have undergone body rotation but not tail retraction. *Pou4* CRISPR served as the “positive control”, eliminating the papilla neurons that trigger metamorphosis (see text for citations). Of the four genes tested, only *Vamp1/2/3* CRISPR appeared to result in substantial loss of tail retraction and body rotation, though not as penetrant as the *Pou4* CRISPR. *n*=100 for each gene. (C) Representative example of *Vamp1/2/3* CRISPR larva, showing intact tail at 45 hpf.

It was recently shown that knockdown or knockout of the neuronal transcription factor *Pou4* eliminates papilla neuron formation and subsequently, papilla neuron-induced signals for tail retraction and metamorphosis (Sakamoto et al., 2022). Other CRISPR gene knockouts were shown to result in a mixture of tail retraction and body rotation defects during settlement and metamorphosis (Johnson et al., 2023 preprint). We therefore used papilla-specific CRISPR/Cas9-mediated knockout in F0 embryos to test the requirement of our candidate genes in a similar tail retraction assay. We used the *Foxc* promoter (Wagner and Levine, 2012) to drive expression of Cas9 in the anteriormost cells of the neural plate, which gives rise to the entire papilla territory and part of the oral siphon primordium (Fig. 3). Embryos were electroporated with 40 μg/700 µl *Foxc>Cas9,* 10 μg/700 µl *Foxc>H2B::mCherry,* and gene-specific pairs of sgRNA vectors (40 µg/700 µl each sgRNA vector). “Positive control” embryos were electroporated as above, using a previously published pair of sgRNA vectors targeting *Pou4* (Johnson et al., 2023 preprint), and “negative control” embryos were electroporated with 10 µg/700 µl *Foxc>H2B::mCherry* alone. All embryos were raised through larval hatching and settlement, and fixed at 45 h post-fertilization, upon which tail retraction and body rotation were scored (Fig. 4A).

As previously reported, *Pou4* knockout in the papilla territory resulted in frequent block of tail resorption and body rotation compared to the negative control (Fig. 4B). Of the gene-specific CRISPR samples, only *Vamp1/2/3* CRISPR showed a substantial effect on metamorphosis, with only 56% of H2B::mCherry+ individuals having retracted their tails (Fig. 4B,C). This was closer to the *Pou4* CRISPR (20% tail retraction) than to the negative control (97% tail retraction). The effect of *Vamp1/2/3* CRISPR on body rotation was very similar (Fig. 4B,C). An independent replicate of *Vamp1/2/3* CRISPR confirmed this result (Fig. S5). Taken together, these data suggest that knocking out *Vamp1/2/3* in the papilla territory impairs the ability of the larva to trigger the onset of metamorphosis.

### Neural tube-specific knockout of *NARS1* causes neurulation defects

Because *NARS1* is associated with various neurodevelopmental defects in mammals (Wang et al., 2020), NARS1 students also tested the requirement of *NARS1* in *Ciona* neurulation. *NARS1* was targeted in the neurectoderm using *Sox1/2/3>Cas9::GemininN,* and the *Nut>Unc-76::GFP* reporter was used to visualize the central nervous system (Shimai et al., 2010). Embryos were electroporated with 40 μg/700 µl *Sox1/2/3>Cas9::GemininN,* 40 μg/700 µl *Nut>Unc-76::GFP,* and 40 µg/700 µl each of both *NARS1* sgRNA vectors. As a result of *NARS1* CRISPR in the neurectoderm, a high frequency of curled/twisting tails specifically in the CRISPR larvae, but not in the negative control electroporated with *Sox1/2/3>Cas9::GemininN* and *Nut>Unc-76::GFP* alone (Fig. S6). This was scored as well, and curved tails were observed in 24 out of 50 *NARS1* CRISPR larvae (48%), compared to 0 out of 50 negative control larvae (0%). Upwards curvature of the tail is a hallmark of impaired neural tube closure in *Ciona* (Mita and Fujiwara, 2007), suggesting *NARS1* may be required in the neural tube for proper neurulation.

## DISCUSSION

We have described the design and validation of sgRNAs targeting four different genes in *Ciona,* in the context of a university-level laboratory course. Of these, only one gene (*Vamp1/2/3*) was shown to be required for tail retraction and body rotation at the onset of metamorphosis. Although the other CRISPR knockouts did not result in a noticeable metamorphosis defect, our validated sgRNAs may be of use to other *Ciona* researchers studying these genes in other contexts.

Our results do not entirely rule out a role for the other three genes tested. For instance, the sgRNAs targeting the other genes might not be efficient enough to cause mutations at a high enough frequency to result in a noticeable defect in our assay. Additionally, there may be similar genes with overlapping functions that can compensate for the loss of one of them. In fact, another NCS family gene, *KH.C9.113*, was also found to be enriched in the putative papilla neuron cell cluster by scRNAseq (Table S1). Another possibility is that the gene may be required for fine-tuned mechanosensory discernment of settlement substrates in the wild, while in our laboratory assays most larvae eventually retract their tails as long as the papilla neurons retain most of their functions. Future studies using appropriate controls will be needed for a more rigorous quantification of subtle metamorphosis defects. Typically one would compare to animals electroporated with a “control” sgRNA that targets no sequence in the *Ciona* genome, or perform a genetic rescue through resupplying the gene product, typically by expressing a cDNA with silent point mutations in the sgRNA target sequences (Stolfi et al., 2014).

Similarly, a role for Dopamine in regulating *Ciona* metamorphosis cannot be ruled out. Our CRISPR knockouts were limited to the papilla territory (due to the use of the papilla-specific *Foxc* promoter to drive Cas9 expression). *Tyrosine hydroxylase* is strongly expressed by the coronet cells of the larval brain region (Moret et al., 2005), and its requirement in these cells has not been directly tested yet.

The requirement of *Vamp1/2/3* for papilla neuron-mediated tail retraction is not surprising, given its central role in synaptic transmission. Recent studies have demonstrated that mechanosensory papilla neuron activity is necessary for tail retraction and metamorphosis in *Ciona* (Hoyer et al., 2023 preprint; Sakamoto et al., 2022; Wakai et al., 2021). While the papilla neurons are glutamatergic, additional neurotransmitters have been implicated in metamorphosis, including noradrenaline (Kimura et al., 2003), GABA, and gonadotropin-releasing hormone (Hozumi et al., 2020). While additional work will be required to identify the exact neurotransmitter(s) released by the papilla neurons of *Ciona* larvae, our results and methods described here establish a proof-of-principle for future screens for genes involved in their development and function.

## MATERIALS AND METHODS

### *Ciona* handling, fixing, staining, and imaging

*Ciona robusta* (*intestinalis Type A*) were collected by and shipped from San Diego, CA, USA (M-REP). Eggs were fertilized, dechorionated, and electroporated according to published protocols (Christiaen et al., 2009a,b). Embryos were raised at 20°C. Embryos, larvae, and/or juveniles were fixed in MEM-FA solution (3.7% formaldehyde, 0.1 M MOPS pH 7.4, 0.5 M NaCl, 1 mM EGTA, 2 mM MgSO4, 0.1% Triton-X100), rinsed in 1X PBS, 0.4% Triton-X100, 50 mM NH4Cl for autofluorescence quenching, and a final 1X PBS, 0.1% Triton-X100 wash. Specimens were imaged on a Leica DMI8 or Nikon Ti2-U inverted epifluorescence microscope.

### Phylogenetic trees

Protein sequences were aligned using online MAFFT version 7 (Katoh et al., 2019). Phylogenetic trees were assembled in MAFFT also, using default parameters: NJ (conserved sites), JTT substitution model, with heterogeneity among sites ignored (α=infinite) and no bootstrapping. Trees were visualized in MAFFT using Archaeopteryx.js (https://github.com/cmzmasek/archaeopteryx-js). Protein domain analysis was performed using SMART (http://smart.embl-heidelberg.de/) (Letunic et al., 2021).

### CRISPR/Cas9 sgRNA design and validation

Single-chain guide RNA (sgRNA) templates were designed using CRISPOR (Haeussler et al., 2016) (crispor.tefor.net) and synthesized custom-cloned into the U6>sgRNA-F+E vector (Stolfi et al., 2014) by Twist Bioscience (South San Francisco, CA, USA). High Doench ‘16 score, high MIT specificity scores were prioritized, and targets containing known single-nucleotide polymorphisms were avoided. Validation of sgRNAs was performed by co-electroporating 25 μg of *Eef1a>Cas9* (Stolfi et al., 2014) and 75 μg of the sgRNA plasmid, per 700 μl of total electroporation volume. Genomic DNA was extracted from larvae electroporated with a given sgRNA using a QIAamp DNA micro kit (Qiagen). PCR products spanning each target site were amplified from the genomic DNA, with each amplicon 150-450 bp in size. Amplicons were purified using a QIAquick PCR purification kit (Qiagen) and Illumina-sequenced using Amplicon-EZ service from Azenta/Genewiz (New Jersey, USA). Papilla-specific CRISPR knockouts were performed using *Foxc>Cas9,* as previously described (Johnson et al., 2023 preprint). *Sox1/2/3>Cas9::GemininN* was constructed using the *Sox1/2/3* promoter (Stolfi et al., 2014) and the *Cas9::GemininN* as previously published (Johnson et al., 2023 preprint; Song et al., 2022). All sgRNA and primer sequences can be found in the Sequences File. All plasmids available upon request. Detailed tutorials and protocols used for classroom activities can be found at the OSF link: https://osf.io/3fh89/ Please contact the corresponding author to inquire about more detailed modifications to commercial kit manufacturers’ protocols. Approximate costs for custom reagents and services described in Table S2.

## Supplementary Material

10.1242/biolopen.060002_sup1 Supplementary information

## References

[BIO060002C1] Athira, A., Dondorp, D., Rudolf, J., Peytral, O. and Chatzigeorgiou, M. (2022). Comprehensive analysis of locomotion dynamics in the protochordate ciona intestinalis reveals how neuromodulators flexibly shape its behavioral repertoire. *PLoS Biol.* 20, e3001744. 10.1371/journal.pbio.3001744 35925898 PMC9352054

[BIO060002C2] Cao, C., Lemaire, L. A., Wang, W., Yoon, P. H., Choi, Y. A., Parsons, L. R., Matese, J. C., Levine, M. and Chen, K. (2019). Comprehensive single-cell transcriptome lineages of a proto-vertebrate. *Nature* 571, 349-354. 10.1038/s41586-019-1385-y 31292549 PMC6978789

[BIO060002C3] Christiaen, L., Wagner, E., Shi, W. and Levine, M. (2009a). Electroporation of transgenic dnas in the sea squirt ciona. *Cold Spring Harb. Protoc*. 2009, pdb. prot5345. 10.1101/pdb.prot5345 20150092

[BIO060002C4] Christiaen, L., Wagner, E., Shi, W. and Levine, M. (2009b). Isolation of sea squirt (ciona) gametes, fertilization, dechorionation, and development. *Cold Spring Harb. Protoc*. 2009, pdb. prot5344. 10.1101/pdb.prot5344 20150091

[BIO060002C5] Cota, C. D. (2018). Transgenic techniques for investigating cell biology during development. In: *Transgenic Ascidians*, ed. by Yasunori Sasakura, pp. 153-164. Springer. 10.1007/978-981-10-7545-2_14 PMC6373173 29542088

[BIO060002C6] Dason, J. S., Romero-Pozuelo, J., Atwood, H. L. and Ferrús, A. (2012). Multiple roles for frequenin/ncs-1 in synaptic function and development. *Mol. Neurobiol.* 45, 388-402. 10.1007/s12035-012-8250-4 22396213

[BIO060002C7] Gandhi, S., Razy-Krajka, F., Christiaen, L. and Stolfi, A. (2018). Crispr knockouts in ciona embryos. In: *Transgenic Ascidians*, ed. by Yasunori Sasakura, pp. 141-152. Springer. 10.1007/978-981-10-7545-2_13 PMC6061950 29542087

[BIO060002C8] Haeussler, M., Schönig, K., Eckert, H., Eschstruth, A., Mianné, J., Renaud, J.-B., Schneider-Maunoury, S., Shkumatava, A., Teboul, L. and Kent, J. (2016). Evaluation of off-target and on-target scoring algorithms and integration into the guide RNA selection tool crispor. *Genome Biol.* 17, 148. 10.1186/s13059-016-1012-2 27380939 PMC4934014

[BIO060002C9] Horie, R., Hazbun, A., Chen, K., Cao, C., Levine, M. and Horie, T. (2018). Shared evolutionary origin of vertebrate neural crest and cranial placodes. *Nature* 560, 228. 10.1038/s41586-018-0385-7 30069052 PMC6390964

[BIO060002C10] Hoyer, J., Kolar, K., Athira, A., Van Den Burgh, M., Dondorp, D., Liang, Z. and Chatzigeorgiou, M. (2023). Polymodal sensory perception of mechanical and chemical cues drives robust settlement and metamorphosis of a marine pre-vertebrate zooplanktonic larva. *bioRxiv.2023-2007*.

[BIO060002C11] Hozumi, A., Matsunobu, S., Mita, K., Treen, N., Sugihara, T., Horie, T., Sakuma, T., Yamamoto, T., Shiraishi, A. and Hamada, M. (2020). Gaba-induced GnRH release triggers chordate metamorphosis. *Curr. Biol.* 30, 1555-1561.e4. 10.1016/j.cub.2020.02.003 32220316

[BIO060002C12] Johnson, C. J., Razy-Krajka, F. and Stolfi, A. (2020). Expression of smooth muscle-like effectors and core cardiomyocyte regulators in the contractile papillae of ciona. *EvoDevo.* 11, 15. 10.1186/s13227-020-00162-x 32774829 PMC7397655

[BIO060002C13] Johnson, C. J., Razy-Krajka, F., Zeng, F., Piekarz, K. M., Biliya, S., Rothbächer, U. and Stolfi, A. (2023). Specification of distinct cell types in a sensory-adhesive organ for metamorphosis in the ciona larva. *bioRxiv*. 10.1371/journal.pbio.3002555 PMC10962819 38478577

[BIO060002C14] Katoh, K., Rozewicki, J. and Yamada, K. D. (2019). Mafft online service: multiple sequence alignment, interactive sequence choice and visualization. *Brief. Bioinform.* 20, 1160-1166. 10.1093/bib/bbx108 28968734 PMC6781576

[BIO060002C15] Katsuyama, Y., Matsumoto, J., Okada, T., Ohtsuka, Y., Chen, L., Okado, H. and Okamura, Y. (2002). Regulation of synaptotagmin gene expression during ascidian embryogenesis. *Dev. Biol.* 244, 293-304. 10.1006/dbio.2002.0584 11944938

[BIO060002C16] Kim, K., Gibboney, S., Razy-Krajka, F., Lowe, E., Wang, W. and Stolfi, A. (2020). Regulation of neurogenesis by fgf signaling and neurogenin in the invertebrate chordate ciona. *Front. Cell Dev. Biol.* 8, 477. 10.3389/fcell.2020.00477 32656209 PMC7324659

[BIO060002C17] Kimura, Y., Yoshida, M. and Morisawa, M. (2003). Interaction between noradrenaline or adrenaline and the beta 1-adrenergic receptor in the nervous system triggers early metamorphosis of larvae in the ascidian, ciona savignyi. *Dev. Biol.* 258, 129-140. 10.1016/S0012-1606(03)00118-0 12781688

[BIO060002C18] Lemaire, P. (2011). Evolutionary crossroads in developmental biology: the tunicates. *Development* 138, 2143-2152. 10.1242/dev.048975 21558365

[BIO060002C19] Letunic, I., Khedkar, S. and Bork, P. (2021). Smart: recent updates, new developments and status in (2020). *Nucleic Acids Res.* 49, D458-D460. 10.1093/nar/gkaa937 33104802 PMC7778883

[BIO060002C20] Mita, K. and Fujiwara, S. (2007). Nodal regulates neural tube formation in the ciona intestinalis embryo. *Dev. Genes Evol.* 217, 593-601. 10.1007/s00427-007-0168-x 17624550

[BIO060002C21] Moret, F., Christiaen, L., Deyts, C., Blin, M., Joly, J. S. and Vernier, P. (2005). The dopamine–synthesizing cells in the swimming larva of the tunicate ciona intestinalis are located only in the hypothalamus–related domain of the sensory vesicle. *Eur. J. Neurosci.* 21, 3043-3055. 10.1111/j.1460-9568.2005.04147.x 15978015

[BIO060002C22] Nakayama-Ishimura, A., Chambon, J.-P., Horie, T., Satoh, N. and Sasakura, Y. (2009). Delineating metamorphic pathways in the ascidian ciona intestinalis. *Dev. Biol.* 326, 357-367. 10.1016/j.ydbio.2008.11.026 19100250

[BIO060002C23] Razy-Krajka, F., Brown, E. R., Horie, T., Callebert, J., Sasakura, Y., Joly, J.-S., Kusakabe, T. G. and Vernier, P. (2012). Monoaminergic modulation of photoreception in ascidian: evidence for a proto-hypothalamo-retinal territory. *BMC Biol.* 10, 45. 10.1186/1741-7007-10-45 22642675 PMC3414799

[BIO060002C24] Razy-Krajka, F., Lam, K., Wang, W., Stolfi, A., Joly, M., Bonneau, R. and Christiaen, L. (2014). Collier/olf/ebf-dependent transcriptional dynamics control pharyngeal muscle specification from primed cardiopharyngeal progenitors. *Dev. Cell* 29, 263-276. 10.1016/j.devcel.2014.04.001 24794633 PMC4045103

[BIO060002C25] Rizo, J. (2022). Molecular mechanisms underlying neurotransmitter release. *Annu. Rev. Biophys.* 51, 377-408. 10.1146/annurev-biophys-111821-104732 35167762 PMC9490555

[BIO060002C26] Sakamoto, A., Hozumi, A., Shiraishi, A., Satake, H., Horie, T. and Sasakura, Y. (2022). The trp channel pkd2 is involved in sensing the mechanical stimulus of adhesion for initiating metamorphosis in the chordate ciona. *Dev. Growth Differ.* 64, 395-408. 10.1111/dgd.12801 36053743

[BIO060002C27] Sasakura, Y. and Horie, T. (2023). Improved genome editing in the ascidian ciona with crispr/cas9 and talen. In: *Genome Editing in Animals: Methods and Protocols* (eds Y. Sasakura and T. Horie), pp. 375-388. USA: Springer. 10.1007/978-1-0716-3016-7_28 36773161

[BIO060002C28] Sharma, S., Wang, W. and Stolfi, A. (2019). Single-cell transcriptome profiling of the ciona larval brain. *Dev. Biol.* 448, 226-236. 10.1016/j.ydbio.2018.09.023 30392840 PMC6487232

[BIO060002C29] Shiba, K., Motegi, H., Yoshida, M. and Noda, T. (1998). Human asparaginyl-trna synthetase: molecular cloning and the inference of the evolutionary history of asx-trna synthetase family. *Nucleic Acids Res.* 26, 5045-5051. 10.1093/nar/26.22.5045 9801298 PMC147956

[BIO060002C30] Shimai, K., Kitaura, Y., Tamari, Y. and Nishikata, T. (2010). Upstream regulatory sequences required for specific gene expression in the ascidian neural tube. *Zoolog. Sci.* 27, 76-83. 10.2108/zsj.27.76 20141411

[BIO060002C31] Song, M., Yuan, X., Racioppi, C., Leslie, M., Stutt, N., Aleksandrova, A., Christiaen, L., Wilson, M. D. and Scott, I. C. (2022). Gata4/5/6 family transcription factors are conserved determinants of cardiac versus pharyngeal mesoderm fate. *Sci. Adv.* 8, eabg0834. 10.1126/sciadv.abg0834 35275720 PMC8916722

[BIO060002C32] Stolfi, A., Gandhi, S., Salek, F. and Christiaen, L. (2014). Tissue-specific genome editing in ciona embryos by crispr/cas9. *Development* 141, 4115-4120. 10.1242/dev.114488 25336740 PMC4302896

[BIO060002C33] Takamura, K., Minamida, N. and Okabe, S. (2010). Neural map of the larval central nervous system in the ascidian ciona intestinalis. *Zoolog. Sci.* 27, 191-203. 10.2108/zsj.27.191 20141424

[BIO060002C34] Thurtle-Schmidt, D. M. and Lo, T. W. (2018). Molecular biology at the cutting edge: a review on crispr/cas9 gene editing for undergraduates. *Biochem. Mol. Biol. Educ.* 46, 195-205. 10.1002/bmb.21108 29381252 PMC5901406

[BIO060002C35] Wagner, E. and Levine, M. (2012). Fgf signaling establishes the anterior border of the ciona neural tube. *Development* 139, 2351-2359. 10.1242/dev.078485 22627287 PMC6514301

[BIO060002C36] Wakai, M. K., Nakamura, M. J., Sawai, S., Hotta, K. and Oka, K. (2021). Two-round ca2+ transient in papillae by mechanical stimulation induces metamorphosis in the ascidian ciona intestinalis type a. *Proc. R. Soc. B.* 288, 20203207. 10.1098/rspb.2020.3207 PMC7935057 33593191

[BIO060002C37] Wang, L., Li, Z., Sievert, D., Smith, D. E. C., Mendes, M. I., Chen, D. Y., Stanley, V., Ghosh, S., Wang, Y., Kara, M. et al. (2020). Loss of nars1 impairs progenitor proliferation in cortical brain organoids and leads to microcephaly. *Nat. Commun.* 11, 4038. 10.1038/s41467-020-17454-4 32788587 PMC7424529

[BIO060002C38] Zega, G., Pennati, R., Groppelli, S., Sotgia, C. and De Bernardi, F. (2005). Dopamine and serotonin modulate the onset of metamorphosis in the ascidian phallusia mammillata. *Dev. Biol.* 282, 246-256. 10.1016/j.ydbio.2005.03.012 15936344

[BIO060002C39] Zeng, F., Wunderer, J., Salvenmoser, W., Ederth, T. and Rothbächer, U. (2019a). Identifying adhesive components in a model tunicate. *Philos. Trans. R. Soc. B.* 374, 20190197. 10.1098/rstb.2019.0197 PMC6745482 31495315

[BIO060002C40] Zeng, F., Wunderer, J., Salvenmoser, W., Hess, M. W., Ladurner, P. and Rothbächer, U. (2019b). Papillae revisited and the nature of the adhesive secreting collocytes. *Dev. Biol.* 448, 183-198. 10.1016/j.ydbio.2018.11.012 30471266

