# Supplementary material for: Using CRISPR/Cas9 to identify genes required for mechanosensory neuron development and function
Source: Biol Open. 2023 Sep 5;12(9):bio060002. doi: 10.1242/bio.060002 (PMC10497037; doi:10.1242/bio.060002)
Supplement: Supplementary information [file biolopen-12-060002-s1.pdf]

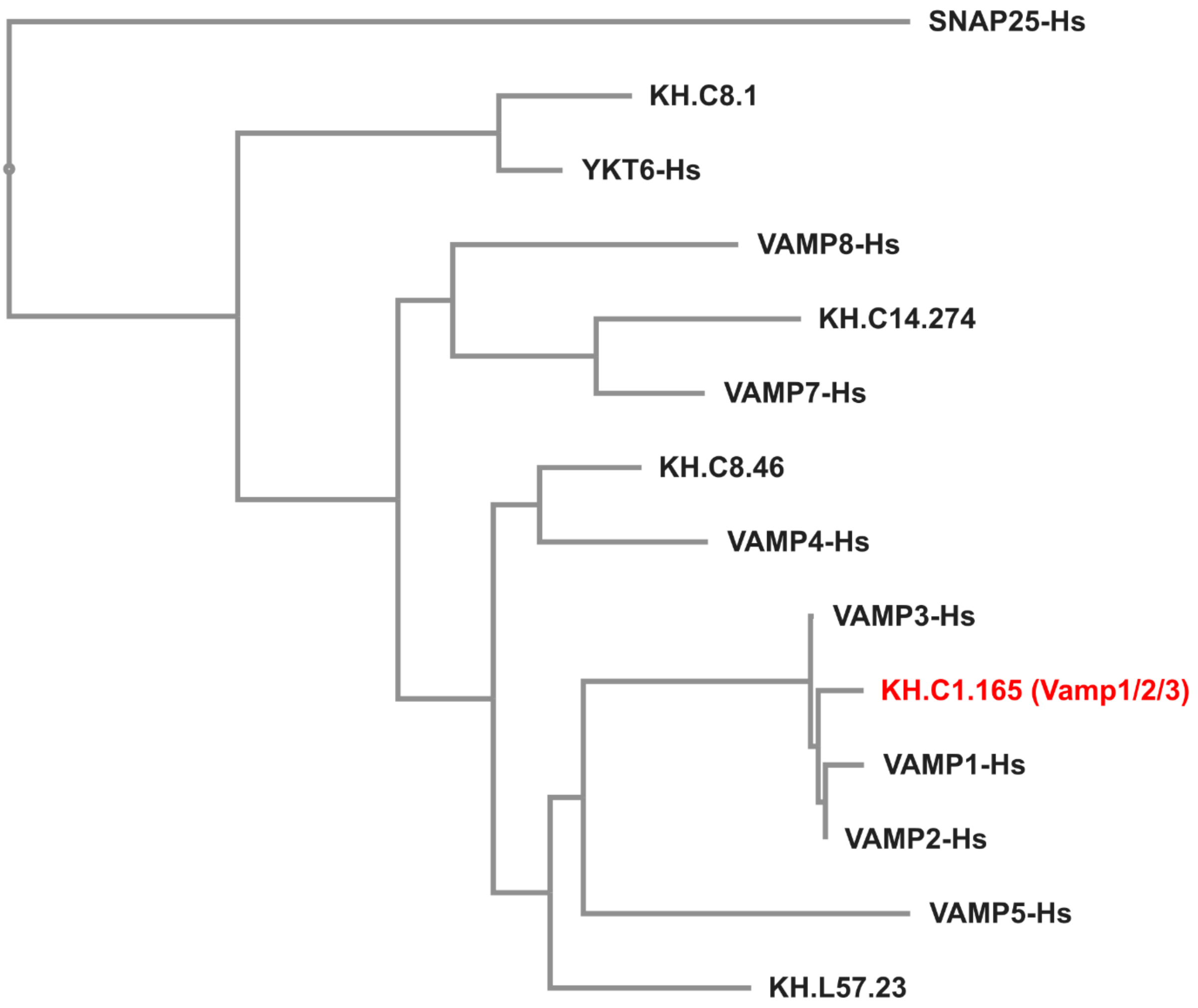


**Fig. S1. Phylogenetic tree of Vamp proteins.**

Tree showing phylogenetic analysis of predicted proteins encoded by *VAMP* family genes from human (Hs) and *Ciona robusta* (KH gene models). See methods for details and supplemental sequences file for protein sequences used.

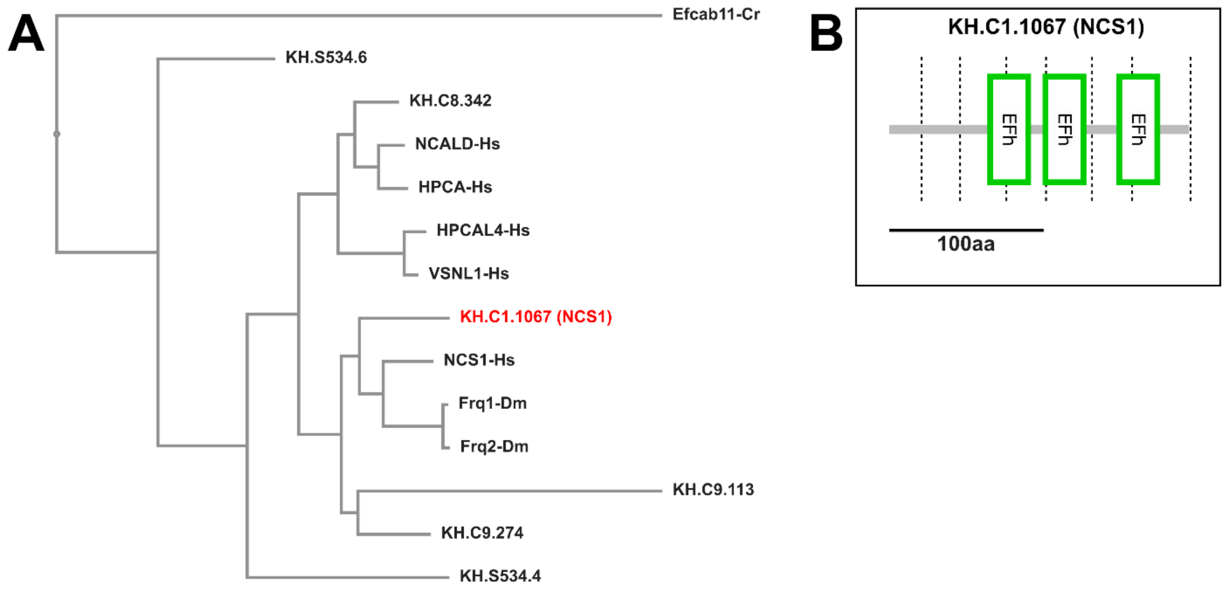


**Fig. S2. Phylogenetic tree of NCS proteins and diagram of *Ciona robusta* NCS1 domains.**

A) Tree showing phylogenetic analysis of proteins encoded by *Neuronal Calcium Sensor (NCS)* family genes from human (Hs), *Drosophila melanogaster* (Dm) and *Ciona robusta* (KH gene models). Efcab11 from *C. robusta* (Cr) was used to root the tree. See methods for details and supplemental sequences file for protein sequences used. B) Protein domain analysis diagram of *Ciona robusta* NCS1 from SMART (Letunic et al. 2021) showing its predicted three EF-hand (Efh) domains. Dashed lines indicate exon-exon junctions.

Biology Open • Supplementary information

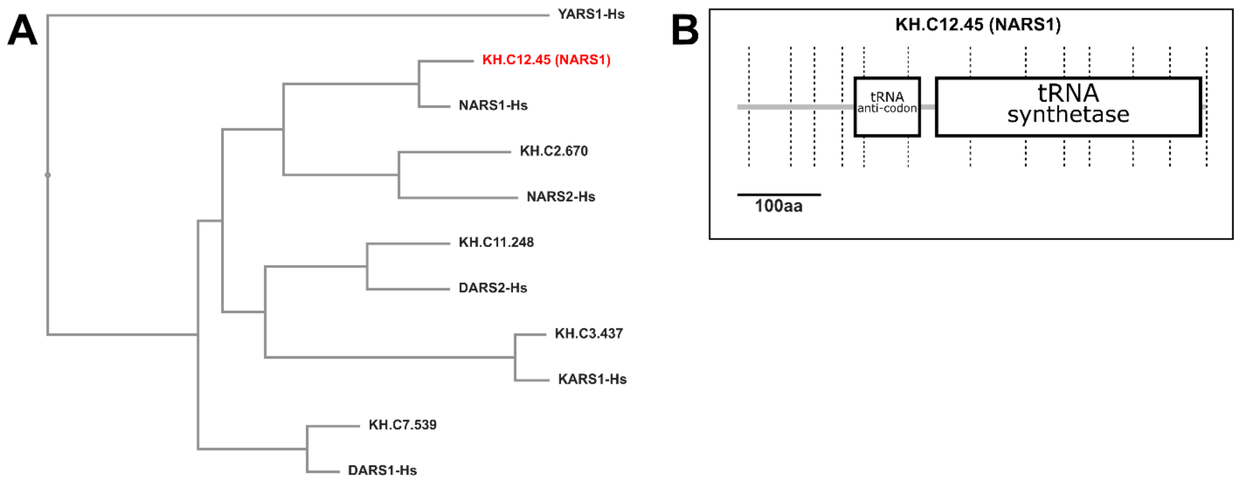


**Fig. S3. Phylogenetic tree of select aminoacyl-tRNA synthetases and *Ciona* NARS1 domains.**

A) Tree showing phylogenetic analysis of proteins encoded by a sampling of aminoacyl-tRNA synthetase genes from human (Hs) and *Ciona robusta* (KH gene models). See methods for details and supplemental sequences file for protein sequences used. B) Protein domain analysis diagram of *Ciona robusta* NARS1 from SMART (Letunic et al. 2021) showing its predicted domains. Dashed lines indicate exon-exon junctions.

Biology Open • Supplementary information

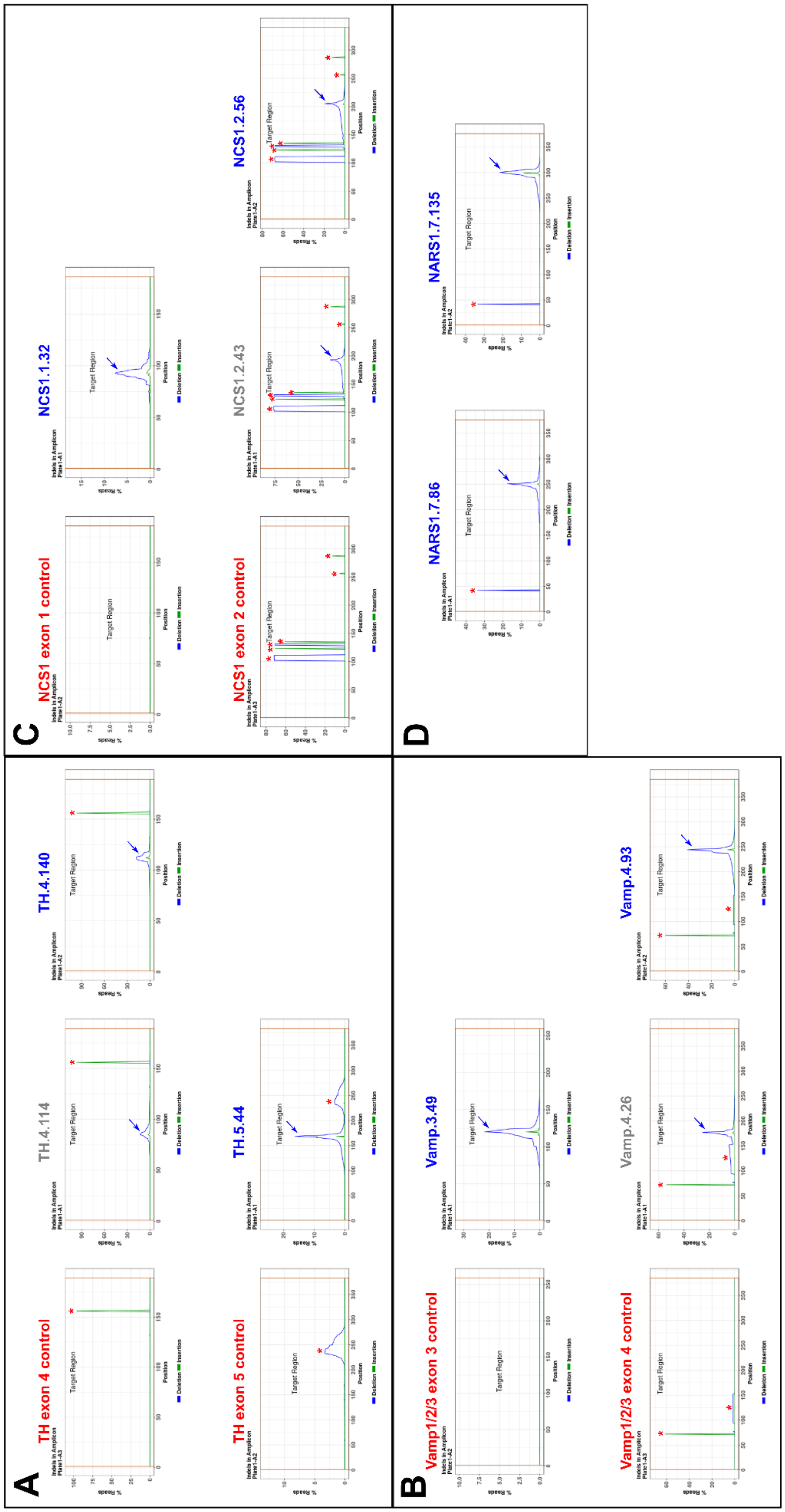
A
TH exon 4 control
TH.4.114
TH.4.140
TH exon 5 control
TH.5.44
B
Vamp1/2/3 exon 3 control
Vamp.3.49
Vamp1/2/3 exon 4 control
Vamp.4.26
Vamp.4.93
C
NCS1 exon 1 control
NCS1.1.32
NCS1 exon 2 control
NCS1.2.43
NCS1.2.56
D
NARS1.7.86
NARS1.7.135
Indels in Amplicon
Target Region
% Reads
Position
Deletion
Insertion

**Fig. S4. Indel plots for all sgRNAs tested.**

A-D) NGS indel validation plots (including negative controls) for all the sgRNAs tested in this study. No amplicon was obtained for the third *NARS1* sgRNA nor the *NARS1* negative control. Blue arrows indicate CRISPR-generated indels, red asterisks indicate naturally occurring indels.

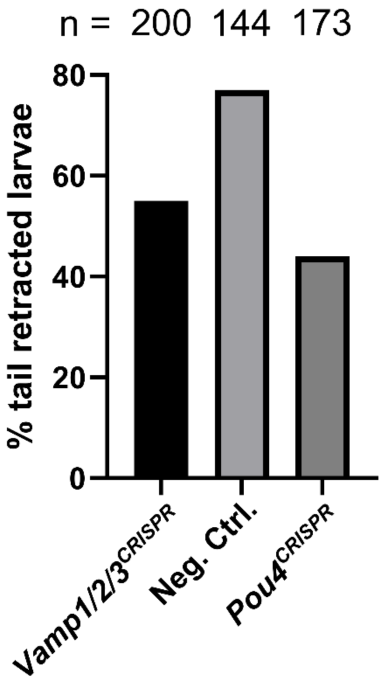


**Fig. S5. Replicate of *Vamp1/2/3* CRISPR.**

Independent replicate of papilla-specific *Vamp1/2/3* CRISPR in *Ciona* larvae. Embryos were electroporated with 40 µg/700 µl *Foxc>Cas9* and gene-specific pairs of sgRNA vectors (40 µg/700 µl each sgRNA vector). Negative control embryos were electroporated with 40 µg/700 µl *Foxc>Cas9* alone. Tail retraction was scored at 48 hours post-fertilization without screening for mCherry+ individuals.

Biology Open • Supplementary information

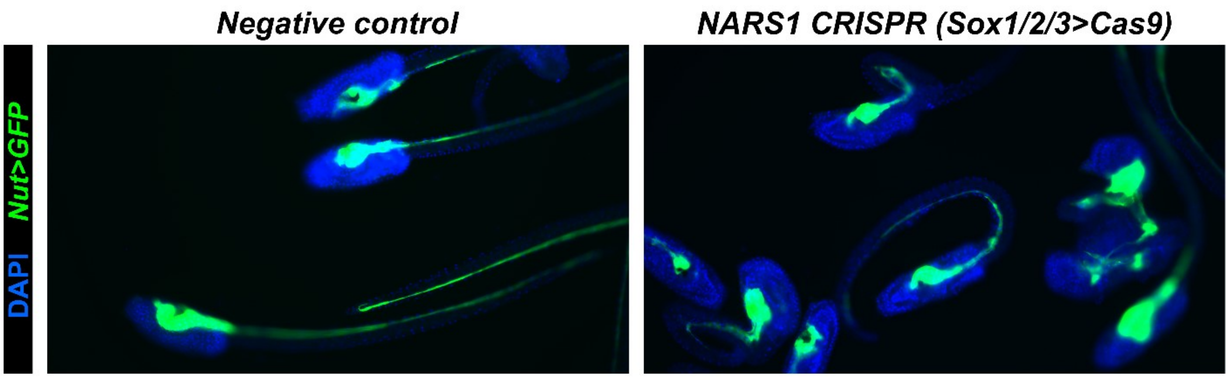


**Fig. S6. Neurectoderm-specific knockout of *NARS1* impairs neurulation.**

Negative control larvae showing normal neural tube and tail morphogenesis, compared to *NARS1* CRISPR larvae. *Nut>Unc-76::GFP* (green) labels the central nervous system. Nuclei counterstained by DAPI (blue). See text for quantification, and for detailed amounts of plasmids used. For approximate sense of scale, larvae can be compared to that in Figure 4C.

**Table S1.**

Click here to download Table S1

**Table S2.** Approximate costs of CRISPR course activities (based on USA pricing). Price for custom sgRNA plasmid (*) can be much lower if using cloning methods described in Stolfi et al. 2014 or Gandhi et al. 2018. Costs of basic reagents for embryo dechorionation, electroporation, fixing, etc. not included.

| Reagent or service | Supplier | Approx. price per sgRNA |
|---|---|---|
| Custom sgRNA plasmid | Twist Bioscience | $120* |
| Maxiprep | Macherey-Nagel (Takara) | $9 |
| Sanger plasmid sequencing | Eurofins | $6 |
| Whole-plasmid sequencing | Plasmidsaurus | $15 |
| Primers for NGS assay | Eurofins | $6 |
| Amplicon sequencing | Azenta | $84 |

Biology Open • Supplementary information

## Supplemental sequences file – Johnson et al. 2023 (Using CRISPR/Cas9 to...)

>Nut -1155/-1 (Nut promoter based on Shimai et al. 2010)
(Sequence of the Nut driver based on genome assembly, not verified)
atctgttctaggaatcttttaactcggcgagcctgtgttttctttttagactataataccctccaataac
agggtaagatgcatgcgaaaaaaacgattgattttgtgcatataaaatatttataatagtctggggttat
gtgagtgaaatagctggttatatctaactgcagccggaaatacgtgctctttgcagaaaggaaaatccat
ttctcgtgtattgcaacatacggtatagtaggtttagatgggtcacctttaccacaaataacatccaaat
atcctgagcatgttttaaacatttaacaacggtatatgtggaagttgcgagaatacggtttgataattca
ttaaatatgctttgtttactacaaaatgggacgggaaaataaaataaaaaggtgtcctatcttcccccat
cctactatatatatcatattttctaatcctgttttagcaattaacaacgctcgtttaaagtcgtgggtat
atgcggttacataaatttaaaaatattatttgtttacttcaaaatagaacgataaaagcgtgtataaaca
tgttgtatcatcttaccccatcctgctacaaatataaacaatttcatgataagaaaatgcacatgtttgt
tatgccgcgtgttgtgtacagttctggttaggatttcagaacacacacacagaaaatcggtcgtgcctgc
tgctgcaactagtgcgacacctcaataattatgagactgcctcagtgcattcctatcaacgtgttttgcg
ctcgtatcacccaaaaagcgtgcctcgtgctgcctacacggtgtggctacataaaaacgtcgcgtgtttt
actgttccagttcgtttttgtattcgtggaaataggttcgcatttttttattaagaaagagtttggttttc
gaggtcactgaattgcaattagaaggcatttaatagtagcactaggacactctattcactgcgttgtcta
aatccatgcgaaacaacaaaataagtcgcaagacatgctgtgcgtgtattgtttttttgaaaccccgtcg
ctttgtgaaaatctggttgattatttttttcgtaccagttttacagtttaaatacgactgtgcttcagttt
ttgttagtattgagttgtacactatatcaacaac

**sgRNAs used in this study:**

TH.4.114
**GCTGGAATCAATTGGTTGCA (G+N19)**

TH.4.140
**GAATTGTTAACGGGACTGAA (G+N19)**

TH.5.44
**GCTGAGCTTGAATTGTGCAG (G+N19)**

Vamp.3.49
**GGTCCAGCTGCACCAACTGG (G+N19)**

Vamp.4.26
**GGACATCATGAGGACAAATG (G+N19)**

Vamp.4.93
**GGGGCAGATGCATTACAGCA (G+N19)**

NCS1.1.32
**GGGCAGCAAACTGAGCGCAG (G+N19)**

NCS1.2.43
**GTGGTATCGAGACTTTATGA (G+N19)**

NCS1.2.56
**GTTATGAAGGATTGTCCCAA (G+N19)**

NARS1.4.25
**GAGAACAAGAAGATGCGGAG (G+N19)**

NARS1.7.86
**GATAGATACAGTCCTCAACC (G+N19)**

NARS1.7.135
**GACAGACATCTAGTTATCCG (G+N19)**

Pou4.3.21 (Johnson et al. 2023)
**GCTGAGTGGTGGAAAGCGGG (G+N19)**

Pou4.4.106 (Johnson et al. 2023)
**GAGGATGGAAATGATTCGGG (G+N19)**

*Primers for amplicon sequencing to measure mutagenesis efficacy*

| Gene + exon | Forward primer | Reverse primer |
|---|---|---|
| TH exon 4 | ACACTAATTAAAAACGTTGCCG | CTATATGTGCCAATAGTGCCC |
| TH exon 5 | TGGGCACTATTGGCACATA | ACATAAATGACGGTAGAATACCC |
| Vamp1/2/3 exon 3 | GCACTTAAACCCTTATATCCCTG | CGATTTTTGACCCGAAGAACA |
| Vamp1/3/4 exon 4 | AGAAACCATGGTGTGCCTC | ATAAGCGTCCACCATTTCCC |
| NCS1 exon 1 | GTGATCTTTGTAGGTCTTCTTT | AGTTAGAAACTAAACGACCGC |
| NCS1 exon 2 | CTGGTTGCTCGCTATAAGTAA | CGTATTCTTGCGACTCTTATAGG |
| NARS1 exon 4 | GGCTACCCTGAATAATCAAAACTT | TTAGCCGTTGTACTGAAGTCC |
| NARS1 exon 7 | GTTGGTATGCCCCTAATTACC | CAAAATCACCTCATAATATCCTCG |

**Sequences for Vamp1/2/3 phylogenetic tree:**

>KH.C1.165.v3.A.SL2-1 (Vamp1/2/3)
MSGYDAVPNPRSYGGTSNYGGPAAPTGGSSQASKKLQQTQAQVDDVVDIMRTNVDKVLERDQKLSELDDR
ADALQQGASQFETQAAKLKRKYWWKNCKMWAILIIVILVIIIIIVVSVVTQTQKKT

>KH.C8.1.v3.A.SL2-2
MVKLFSLSLFYKGESCVIPLIAEYDTDSFSFFQRKSVQEFLSFTSKIITERTHPGMRQSVKEQEHLCHVY
VREDRLAAVLISDEDYPRRVAFTVLTKVCEDFAKKFPPSELNNPAPMNIQFEGVREMLCKYQNPKEADSM
SRVQAELDETKIVLHGTMESLLQRGEKLDDLVAKSDQLSSQSKMFYKQIKIHMKHDKHLWKKCTIAYRAN
FHLV

>KH.C14.274.v1.B.ND1-1
MTILFSIIARGTTVLARYASCAGNFQEVSEQILSKITADDAKLTYSHGSYLFHYICDDRIVYMAITEDDF
ERSKAFRYLSDIRKKFQSTYGKNVHTALPYAMDTDFARVLMVQMKRFSSNEEPENKVEEVQDQLNDLKGI
MVKNIDSIANRGENLNLLVDKTEDLSESAVTFKKQSTTLRRRLWWKNVKITVILVIVAIIVLYFIICAAC
GGMNWPRCVHQSNSPNKTLTLT

>KH.L57.23.v2.A.ND1-1
MTDNGLVYLCAADKEFGRRIPYLFLEELKKQFCSSGSLLQRTTGASAFEFNRDFRNILSGIMDDFNKGKG
DQLSTMQNQVGEVTGIMRQNIEKVIERGDKLDDLVDKTEDLQAGAATFKVTAKRIQRKYFWQNKKMLIII
IVIVLIIITLIVLFATGTI

>KH.C8.46.v1.A.ND1-1

Biology Open • Supplementary information

MTRYNKGKNTERVALLDDYESDDDFFLKGPSSASDKVKRVQSQVNEVVDVMQTNIGKVLERGDKLEDLQD
KSESLADSATHFNTHATRLRKKMWWKDMRTKIIIGVLLLMILIIIIVSIAVKNKGS

>VAMP1-Hs
MSAPAQPPAEGTEGTAPGGGPPGPPPNMTSNRRLQQTQAQVEEVVDIIRVNVDKVLERDQKLSELDDRAD
ALQAGASQFESSAAKLKRKYWWKNCKMMIMLGAICAIIVVVIVIYFFT

>VAMP2-Hs
MSATAATAPPAAPAGEGGPPAPPPNLTSNRRLQQTQAQVDEVVDIMRVNVDKVLERDQKLSELDDRADAL
QAGASQFETSAAKLKRKYWWKNLKMMIILGVICAIILIIIIVYFST

>VAMP3-Hs
MSTGPTAATGSNRRLQQTQNQVDEVVDIMRVNVDKVLERDQKLSELDDRADALQAGASQFETSAAKLKRK
YWWKNCKMWAIGITVLVIFIIIIIVWVVSS

>VAMP4-Hs
MPPKFKRHLNDDDVTGSVKSERRNLLEDDSDEEEDFFLRGPSGPRFGPRNDKIKHVQNQVDEVIDVMQEN
ITKVIERGERLDELQDKSESLSDNATAFSNRSKQLRRQMWWRGCKIKAIMALVAAILLLVIIILIVMKYR
T

>VAMP5-Hs
MAGIELERCQQQANEVTEIMRNNFGKVLERGVKLAELQQRSDQLLDMSSTFNKTTQNLAQKKCWENIRYR
ICVGLVVVGVLLIIILIVLLVVFLPQSSDSSSAPRTQDAGIASGPGN

>YKT6-Hs
MKLYSLSVLYKGEAKVVLLKAAYDVSSFSFFQRSSVQEFMTFTSQLIVERSSKGTRASVKEQDYLCHVYV
RNDSLAGVVIADNEYPSRVAFTLLEKVLDEFSKQVDRIDWPVGSPATIHYPALDGHLSRYQNPREADPMT
KVQAELDETKIILHNTMESLLERGEKLDDLVSKSEVLGTQSKAFYKTARKQNSCCAIM

>VAMP7-Hs
MAILFAVVARGTTILAKHAWCGGNFLEVTEQILAKIPSENNKLTYSHGNYLFHYICQDRIVYLCITDDDF
ERSRAFNFLNEIKKRFQTTYGSRAQTALPYAMNSEFSSVLAAQLKHHSENKGLDKVMETQAQVDELKGIM
VRNIDLVAQRGERLELLIDKTENLVDSSVTFKTTSRNLARAMCMKNLKLTIIIIIVSIVFIYIIVSPLCG
GFTWPSCVKK

>VAMP8-Hs
MEEASEGGGNDRVRNLQSEVEGVKNIMTQNVERILARGENLEHLRNKTEDLEATSEHFKTTSQKVARKFW
WKNVKMIVLICVIVFIIILFIVLFATGAFS

>SNAP25-Hs
MAEDADMRNELEEMQRRADQLADESLESTRRMLQLVEESKDAGIRTLVMLDEQGEQLERIEEGMDQINKD
MKEAEKNLTDLGKFCGLCVCPCNKLKSSDAYKKAWGNNQDGVVASQPARVVDEREQMAISGGFIRRVTND
ARENEMDENLEQVSGIIGNLRHMALDMGNEIDTQNRQIDRIMEKADSNKTRIDEANQRATKMLGSG

**Sequences for NCS1 phylogenetic tree:**

>KH.C1.1067.v1.A.SL1-1 (NCS1)
MGKSGSKLSAEDAQTLQTTTHFDKKEIQKWYRDFMKDCPNGYLKKEEFQKVYQQFFPKGNPSKFANFVFN
VFDSDKDGFITFKEFISALSVTSRGNLDEKLDWAFNLYDLDHDGFITREEMLNIVDAIYSMVGNAMDLPE
DENTPEKRVNKIFCQMDQNKDGKLTKDEFREGSKCDPYIVKALSAGLGGAESCPS

Biology Open • Supplementary information

>KH.C9.113.v1.A.ND1-1
MGNQESTQSLSEHEIARLSAETNFTPAEVAQWYKGFKRDCPSGKFTMARFQAIAEGFFPAGDAENFTKFI
FNGMDLEEDASLNFESFIKVTSLLARGTKEEKLKWAFHVRDIDHTGFITKEKMQLVEDSVFSMIANHVPL
PDDENTAEKRTEKLYNLIEKDQDGKVTVDQFKVGLNQDRDIVNALSLHDTVQ

>KH.S534.4.v2.A.SL3-1
MGCAKSKTRDEDCKQLESLTNFTPEELKKCYDDFQQESTSGKMDRTKFDEFYKKFFNRDPRFVDHLFRTF
DFNNDGFINFREFVCGLSITTRGTPEEKLTWTFNVYDVNNDGTITRDEMLQIMRAIYAMNGISEPEQLKS
GSDAFEGLDSNGDGLISVAEFVKGVKRDERLLEFLQRTIDVQQK

>KH.S534.6.v1.A.nonSL2-1
MGQQHPSLKPRMLDDLMRMSDFSADELKIWYNEFSKDSVSGFLSKNDFIKIYKGLFPHGDASGFADHVFR
TFDANKDGVLNFREFVIGLSLTMKGPLDDKLYWAFKLYDVDGNGFVTKDEMNEIIAVSNAIFTVCCTDTT
DDDVIMSGREAFKTMDTDEDGKVSWGEFRKGIHSNRVFMLIVEAKMKNAGVTSR

>KH.C8.342.v1.A.SL2-1
MGKQNSKLKPEDIRDLRAATEFNEHELQEWYKGFIKDCPSGNLTMDEFQKIYANFFPQGDASKFAAHVFR
TFDSNGDSTIDFREFIVALSVTSRGNLEEKLKWAFSMYDCDGNGIISRDEMLEIVRAIYKMVGAVMKMPE
DESTPEKRTDKIFKQMDKNLDGSISLEEFVEGAKKDPSIVRLLQCTPGSGGLGM

>KH.C9.274.v1.A.SL1-1
MGNRKSKLKPEVLEKLTKQTKFTEAELHQWHKGFLHDCPTGKLSYEEFQGIYRQFFPQGNSAKFAKLVFT
TFDDNKDGTVEFEEFIIALSVTSRGTLDEKLHWAFQLYDLDNDGFITKDEMLNIVEAIFAMVGDAVNLPA
EENTPQKRVEKIFKVMDKNKDGKLTKDEFLVGAKSDPSIVQALSIYDGLV

>NCS1-Hs
MGKSNSKLKPEVVEELTRKTYFTEKEVQQWYKGFIKDCPSGQLDAAGFQKIYKQFFPFGDPTKFATFVFN
VFDENKDGRIEFSEFIQALSVTSRGTLDEKLRWAFKLYDLDNDGYITRNEMLDIVDAIYQMVGNTVELPE
EENTPEKRVDRIFAMMDKNADGKLTLQEFQEGSKADPSIVQALSLYDGLV

>NCALD-Hs
MGKQNSKLRPEVMQDLLESTDFTEHEIQEWYKGFLRDCPSGHLSMEEFKKIYGNFFPYGDASKFAEHVFR
TFDANGDGTIDFREFIIALSVTSRGKLEQKLKWAFSMYDLDGNGYISKAEMLEIVQAIYKMVSSVMKMPE
DESTPEKRTEKIFRQMDTNRDGKLSLEEFIRGAKSDPSIVRLLQCDPSSAGQF

>HPCA-Hs
MGKQNSKLRPEMLQDLRENTEFSELELQEWYKGFLKDCPTGILNVDEFKKIYANFFPYGDASKFAEHVFR
TFDTNSDGTIDFREFIIALSVTSRGRLEQKLMWAFSMYDLDGNGYISREEMLEIVQAIYKMVSSVMKMPE
DESTPEKRTEKIFRQMDTNNDGKLSLEEFIRGAKSDPSIVRLLQCDPSSASQF

>HPCAL4-Hs
MGKTNSKLAPEVLEDLVQNTEFSEQELKQWYKGFLKDCPSGILNLEEFQQLYIKFFPYGDASKFAQHAFR
TFDKNGDGTIDFREFICALSVTSRGSFEQKLNWAFEMYDLDGDGRITRLEMLEIIEAIYKMVGTVIMMRM
NQDGLTPQQRVDKIFKKMDQDKDDQITLEEFKEAAKSDPSIVLLLQCDMQK

>VSNL1-Hs

MGKQNSKLAPEVMEDLVKSTEFNEHELKQWYKGFLKDCPSGRLNLEEFQQLYVKFFPYGDASKFAQHAFR
TFDKNGDGTIDFREFICALSITSRGSFEQKLNWAFNMYDLDGDGKITRVEMLEIIEAIYKMVGTVIMMKM
NEDGLTPEQRVDKIFSKMDKNKDDQITLDEFKEAAKSDPSIVLLLQCDIQK

>Frq1-Dm
MGKKSSKLKQDTIDRLTTDTYFTEKEIRQWHKGFLKDCPNGLLTEQGFIKIYKQFFPQGDPSKFASLVFR
VFDENNDGSIEFEEFIRALSVTSKGNLDEKLQWAFRLYDVDNDGYITREEMYNIVDAIYQMVGQQPQSED
ENTPQKRVDKIFDQMDKNHDGKLTLEEFREGSKADPRIVQALSLGGG

>Frq2-Dm
MGKKNSKLKQDTIDRLTTDTYFTEKEIRQWHKGFLKDCPNGLLTEQGFIKIYKQFFPDGDPSKFASLVFR
VFDENNDGAIEFEEFIRALSITSRGNLDEKLHWAFRLYDVDNDGYITREEMYNIVDAIYQMVGQQPQTED
ENTPQKRVDKIFDQMDKNHDDRLTLEEFREGSKADPRIVQALSLGGD

>Efcab11-Cr (KH.C10.577.v1.A.ND1-1)
MAVSLRRLQTIFNHCDEDKKGFLNREDLKVAMLILFGYKPSKYEIQQLLDDGEGNKKLMNFETFKSIMAA
SKHDPDHEIRDIFRMFDTHCRGFLIFDDVKKAFHAVAPHISDATIRACCEEMCRESDGRISYREFAEAMR
HGHVHMEHHENYDHFFNSNVCFS

**Sequences for NARS1 phylogenetic tree:**

>KH.C12.45.v1.A.SL1-1 (NARS1)
MGDSVEQQVNKLSIGELYTSEKTGCDESGDGTEAKPFKTILRAMMFHQSEPFPTLYVDSKEPNKKFDPIA
KAQLKKQTKIFTAEKRKDVARKVREQEDAERREKNLEDAKKIIISEDKSLPAAKSIKIGAATAHRGQRVK
VNGWSHRIRRQGKTLMFIVLRDGSGYLQAVLNDQLCQTYNAVMLSTESTVCLYGVITPVMEGKSAPGGHE
LICDYWELIGLAPPGGIDTVLNQEADVDVMLDNRHLVIRGEQTSKILRVRSYLMQAFRDHYFERGYYEVT
PPCLVQTQCEGGSTLFKLDFFGEQAYLTQSSQLYLETCLPSLGDVFCLAESYRAEQSRTRRHLAEYTHVE
AECAFITFNDLLDRLEDLICDVVDKLLKSPAGELIKDLNPGFVAPKRPFRRMDYAEAIVYLKEHDIKKED
GSYYEFGEDIPEMPERKMTDQINEPILLCRFPYKIKSFYMQKCKDNLELTESVDLLMPNVGEIVGGSMRM
DNHEELLEGYKSEGVDPSKYYWYTDQRKFGTCEHGGYGLGLERLLTWLTDRYHIRDVCLYPRFLERCCP

>KH.C7.539.v1.A.SL1-1
MADVENVPLGDDGKPMSKKALKKQQKDAEKAAKKAQRQQEQADAQQKADADDVSKDLYGDMVMIQSSEKP
DRCLQPIGDIGPNLAEKNIWVRGRLHTSRGTGKQCFTVLRHQQSTIQALIFVGEKISKQMVKFCANISKE
SIVDVYGVVKKVEQKVESCTQDDVEIHIGKIFVVSKAAPQLPLLIEDASRPELEDGKNEEGGRPRVNLDT
RLDNRILDLRTTTKQAIFRLEAGVCKLFRDTLTAKGFCEIHTPKIISAASEGGANVFTVSYFKTNAFLAQ
SPQLYKQMAIAADFEKVFTVGAVFRAEDSNTHRHLTEFVGLDLEMAFQYHYHEVLQVIGDMFISIFKGLR
ESYQKEITTVARQYPAEPFKFLEPTLILQYPEAVKMLNDAGVEMQDNEDLSTPSEKLLGRLVRQKYDTDY
YILDKYPLEVRPFYTMPDPDNPKWSNSYDMFMRGEEILSGAQRIHDPEFLSERAKAHGIDLKTIEAYIDS
FKYGCPPHAGGGIGMERVVMLYLGLHNIRSSSLFPRDPKRLTP

>KH.C2.670.v1.A.ND1-1
MFRLCKLSYSTVTTALKRFKIKDVVNGEAVSQNGWIQGWVQSIRKHKNFMFVDIVDGTSLNPLQVVLPSH
MFNSQLQNGAAVSANGTFVETDLNVNKIEMVCEEIQVVGPCDPETYPFLKHTPHDMQHLRSYPHLRMRRT
SMINAFKLRNQLEMLFHEFFQNEGFTHVHTPIITLSDCEGAGELFSIKSDTSTNTDMENSENHFFNKPAY
LTVSGQMHLEACAMSLGSVYTLSPVFRAEHGISRKHLSEFRMLEVEVAFTQDLHEILDLIEDSIKFCIER
VQSVPLKKFDDGYKDIPPQHHKAVKNATCSNYVRIPYSEALEILQKNSHKLTTAAPVWGDDFNTDQESFL
VKHFGLTPVFIINFPKVAKPFYMYANNDNNTVAAVDLIFPFCGEICGGSLREHRLELLQDSLRTSGLNEK
DYEWYLDLRRFGSAPHGGYGLGFDRLVHFLLGTRNIKDAVPFPRTPHSCPL

Biology Open • Supplementary information

>KH.C3.437.v2.A.SL1-1
MADNGDCEEKKISKNEMKRQLKAQQKAKEKAEKAAKALENAPKEVKPEKTASKEEETLDPNQYLQIRKNT
ITTLRQNNIEPYPHKFHVSISLSDYVEKYNNIEVGSHLNDQQVSIAGRIHAKREAGPKLIFYDVRGDGVK
LQVMANSKMYSSEEAYQEINERTRRGDIIGVIGHPAKTKKGELSIVPNTIEILSPCLHMLPHLHFGLKDK
ETRYRQRYLDLIMNDQTRQKFITRAKIISYIRSFFDQMGFLEVETPMMNMVAGGATAKPFITHHNDLDMD
LYMRVAPELYLKMLVVGGLDRVYEIGRLFRNEGIDMTHNPEFTSCEFYMAYADYEDLMKISETLISGMVK
QICGSYKLTYHPDGPDSEGYEVDYTPPFRRLRMLPDLERLTGMEFPKPTELHTAGAQARLDEICVKLGVE
CPPPRTTARLLDKLVGDYLEVNCINPTFITEHPEIMSPLAKWHRSIKGLTERFELFVNKKEICNAYTELN
DPMIQRQRFEQQALDKAAGDDEAQMVDENFCTALEYGLPPTGGWGMGIDRLTMFLTDSNNIKEVLLFPAM
KPDDQVPKKAEDSPST

>KH.C11.248.v1.A.SL2-1
MLKLFRVVRPILFKFRPKFNPVTICIVTKQFTSNSAIPQVVQSIKNEKLPNSKFSYRSHTCGELRIKNAG
EAVSLCGWLEYKRKDFLVLRDSFGSVQILITGYEDKNQTFQNVKPESVVKVTGKVTTRPEGQANPNMITG
EIEIIPDNIEILNKCKFLPFRVQKFAGKGERIRQKYRYLDIRGDILQQNLRLRSKVLSKMRKSLEENCFV
EVETPTLFHPTPGGAREFIVPSSSNPGKFYSLPQSPQQLKQLLMIGGVDRYYQFARCYRDEPFKRDRQVE
FTQLDIEMSFVDVNDVIKLSEEVIKSSWPRKFSDESFKLITYDDAMSKYGSDKPDTRFGMLLHDITTFIP
DTLVELFQGDHSDPVNIQAVVVENAQKHFSSKFRRYIGSRMTSAIDDPSPYALLGLVNGQWSGYHKNTNI
GSMVTKQLVDHLQLGGSDIVVVSWGEKRSAQKTLGALRRIISRIFDEISVKHRNVDLDQFLWVVDFPLFE
ADPYTGSLCTVHHPFTSPHPDDVHLLEVDPLKVRSQAYDLVLNGNEVGGGSIRIHDSKLQRQIFTLLNLD
FTELSYLLEALNSGAPPHGGIAFGIDRIMSILCGTETIRDVIAFPKNSRGEDLMTNSPISINSNILKEFH
IKTNVSDK

>YARS1-Hs
MGDAPSPEEKLHLITRNLQEVLGEEKLKEILKERELKIYWGTATTGKPHVAYFVPMSKIADFLKAGCEVT
ILFADLHAYLDNMKAPWELLELRVSYYENVIKAMLESIGVPLEKLKFIKGTDYQLSKEYTLDVYRLSSVV
TQHDSKKAGAEVVKQVEHPLLSGLLYPGLQALDEEYLKVDAQFGGIDQRKIFTFAEKYLPALGYSKRVHL
MNPMVPGLTGSKMSSSEEESKIDLLDRKEDVKKKLKKAFCEPGNVENNGVLSFIKHVLFPLKSEFVILRD
EKWGGNKTYTAYVDLEKDFAAEVVHPGDLKNSVEVALNKLLDPIREKFNTPALKKLASAAYPDPSKQKPM
AKGPAKNSEPEEVIPSRLDIRVGKIITVEKHPDADSLYVEKIDVGEAEPRTVVSGLVQFVPKEELQDRLV
VVLCNLKPQKMRGVESQGMLLCASIEGINRQVEPLDPPAGSAPGEHVFVKGYEKGQPDEELKPKKKVFEK
LQADFKISEECIAQWKQTNFMTKLGSISCKSLKGGNIS

>DARS1-Hs
MPSASASRKSQEKPREIMDAAEDYAKERYGISSMIQSQEKPDRVLVRVRDLTIQKADEVVWVRARVHTSR
AKGKQCFLVLRQQQFNVQALVAVGDHASKQMVKFAANINKESIVDVEGVVRKVNQKIGSCTQQDVELHVQ
KIYVISLAEPRLPLQLDDAVRPEAEGEEEGRATVNQDTRLDNRVIDLRTSTSQAVFRLQSGICHLFRETL
INKGFVEIQTPKIISAASEGGANVFTVSYFKNNAYLAQSPQLYKQMCICADFEKVFSIGPVFRAEDSNTH
RHLTEFVGLDIEMAFNYHYHEVMEEIADTMVQIFKGLQERFQTEIQTVNKQFPCEPFKFLEPTLRLEYCE
ALAMLREAGVEMGDEDDLSTPNEKLLGHLVKEKYDTDFYILDKYPLAVRPFYTMPDPRNPKQSNSYDMFM
RGEEILSGAQRIHDPQLLTERALHHGIDLEKIKAYIDSFRFGAPPHAGGGIGLERVTMLFLGLHNVRQTS
MFPRDPKRLTP

>DARS2-Hs
MYFPSWLSQLYRGLSRPIRRTTQPIWGSLYRSLLQSSQRRIPEFSSFVVRTNTCGELRSSHLGQEVTLCG
WIQYRRQNTFLVLRDFDGLVQVIIPQDESAASVKKILCEAPVESVVQVSGTVISRPAGQENPKMPTGEIE
IKVKTAELLNACKKLPFEIKNFVKKTEALRLQYRYLDLRSFQMQYNLRLRSQMVMKMREYLCNLHGFVDI
ETPTLFKRTPGGAKEFLVPSREPGKFYSLPQSPQQFKQLLMVGGLDRYFQVARCYRDEGSRPDRQPEFTQ
IDIEMSFVDQTGIQSLIEGLLQYSWPNDKDPVVVPFPTMTFAEVLATYGTDKPDTRFGMKIIDISDVFRN
TEIGFLQDALSKPHGTVKAICIPEGAKYLKRKDIESIRNFAADHFNQEILPVFLNANRNWNSPVANFIME
SQRLELIRLMETQEEDVVLLTAGEHNKACSLLGKLRLECADLLETRGVVLRDPTLFSFLWVVDFPLFLPK
EENPRELESAHHPFTAPHPSDIHLLYTEPKKARSQHYDLVLNGNEIGGGSIRIHNAELQRYILATLLKED

VKMLSHLLQALDYGAPPHGGIALGLDRLICLVTGSPSIRDVIAFPKSFRGHDLMSNTPDSVPPEELKPYH
IRVSKPTDSKAERAH

>NARS1-Hs
MVLAELYVSDREGSDATGDGTKEKPFKTGLKALMTVGKEPFPTIYVDSQKENERWNVISKSQLKNIKKMW
HREQMKSESREKKEAEDSLRREKNLEEAKKITIKNDPSLPEPKCVKIGALEGYRGQRVKVFGWVHRLRRQ
GKNLMFLVLRDGTGYLQCVLADELCQCYNGVLLSTESSVAVYGMLNLTPKGKQAPGGHELSCDFWELIGL
APAGGADNLINEESDVDVQLNNRHMMIRGENMSKILKARSMVTRCFRDHFFDRGYYEVTPPTLVQTQVEG
GATLFKLDYFGEEAFLTQSSQLYLETCLPALGDVFCIAQSYRAEQSRTRRHLAEYTHVEAECPFLTFDDL
LNRLEDLVCDVVDRILKSPAGSIVHELNPNFQPPKRPFKRMNYSDAIVWLKEHDVKKEDGTFYEFGEDIP
EAPERLMTDTINEPILLCRFPVEIKSFYMQRCPEDSRLTESVDVLMPNVGEIVGGSMRIFDSEEILAGYK
REGIDPTPYYWYTDQRKYGTCPHGGYGLGLERFLTWILNRYHIRDVCLYP
RFVQRCTP

>NARS2-Hs
MLGVRCLLRSVRFCSSAPFPKHKPSAKLSVRDALGAQNASGERIKIQGWIRSVRSQKEVLFLHVNDGSSL
ESLQVVADSGLDSRELNFGSSVEVQGQLIKSPSKRQNVELKAEKIKVIGNCDAKDFPIKYKERHPLEYLR
QYPHFRCRTNVLGSILRIRSEATAAIHSFFKDSGFVHIHTPIITSNDSEGAGELFQLEPSGKLKVPEENF
FNVPAFLTVSGQLHLEVMSGAFTQVFTFGPTFRAENSQSRRHLAEFYMIEAEISFVDSLQDLMQVIEELF
KATTMMVLSKCPEDVELCHKFIAPGQKDRLEHMLKNNFLIISYTEAVEILKQASQNFTFTPEWGADLRTE
HEKYLVKHCGNIPVFVINYPLTLKPFYMRDNEDGPQHTVAAVDLLVPGVGELFGGGLREERYHFLEERLA
RSGLTEVYQWYLDLRRFGSVPHGGFGMGFERYLQCILGVDNIKDVIPFPRFPHSCLL

>KARS1-Hs
MAAVQAAEVKVDGSEPKLSKNELKRRLKAEKKVAEKEAKQKELSEKQLSQATAAATNHTTDNGVGPEEES
VDPNQYYKIRSQAIHQLKVNGEDPYPHKFHVDISLTDFIQKYSHLQPGDHLTDITLKVAGRIHAKRASGG
KLIFYDLRGEGVKLQVMANSRNYKSEEEFIHINNKLRRGDIIGVQGNPGKTKKGELSIIPYEITLLSPCL
HMLPHLHFGLKDKETRYRQRYLDLILNDFVRQKFIIRSKIITYIRSFLDELGFLEIETPMMNIIPGGAVA
KPFITYHNELDMNLYMRIAPELYHKMLVVGGIDRVYEIGRQFRNEGIDLTHNPEFTTCEFYMAYADYHDL
MEITEKMVSGMVKHITGSYKVTYHPDGPEGQAYDVDFTPPFRRINMVEELEKALGMKLPETNLFETEETR
KILDDICVAKAVECPPPRTTARLLDKLVGEFLEVTCINPTFICDHPQIMSPLAKWHRSKEGLTERFELFV
MKKEICNAYTELNDPMRQRQLFEEQAKAKAAGDDEAMFIDENFCTALEYGLPPTAGWGMGIDRVAMFLTD
SNNIKEVLLFPAMKPEDKKENVATTDTLESTTVGTSV

Cr: Ciona robusta
Hs: Homo sapiens
Dm: Drosophila melanogaster
